# Focus on the Avalanche Breakdown Characteristic of Si- and InP-Based APDs Irradiated by Fast Neutrons

**DOI:** 10.3390/mi14010086

**Published:** 2022-12-29

**Authors:** Jianbin Kang, Qian Li, Xiang Fu, Feiliang Chen, Mo Li

**Affiliations:** 1 Microsystem and Terahertz Research Center of Institute of Electronic Engineering, China Academy of Engineering Physics, Chengdu 610200, China; 2 Key Laboratory of Optoelectronic Technology and Systems, Ministry of Education, Chongqing University, Chongqing 400044, China; 3 School of Electronic Science and Engineering, University of Electronic Science and Technology of China, Chengdu 610054, China

**Keywords:** avalanche breakdown, fast neutron, APD

## Abstract

The Si- and InP-based APDs as the most important weak light semiconductor photodetectors to have achieved commercial success and are widely used in irradiation environments. Investigating the influencing mechanism of neutron irradiation on the above two types of APDs is of scientific and practical importance. In this paper, the dark current and gain characteristics of Si- and InP-based APDs around breakdown voltage were analyzed in detail before and after irradiation. The increase of dark current and the decrease of gain were observed for both the neutron irradiated Si- and InP-based APDs. Generation centers induced by neutrons are responsible for the increased dark current. The decrease of gain can be attributed to the increase of multiplied dark current and the change of electric field distribution in APD. The Si-based APD exhibits soft breakdown with the breakdown voltage reduced by ~8 V under the neutron fluence of 1.0 × 10^12^ cm^−2^, while the soft breakdown occurs along with a small change of breakdown voltage of ~1.5 V under the neutron fluence of 1.0 × 10^13^ cm^−2^ for InP-based APD. The difference in the change of breakdown voltage probably occurs because the Si-based APD uses p-doped Si as the multiplication layer, in which the neutron induced carrier removing effect cannot be ignored to keep the electric field distribution away from the optimal state. Therefore, using an intrinsic multiplication layer in APD is helpful to improve the neutron radiation resistance. The findings here are not only useful for the radiation hardened design of APD, but also deepen the understanding of irradiation mechanism.

## 1. Introduction

Avalanche photodiode (APD) is a kind of semiconductor photodetector with internal gain, which has become a research hotspot in the field of weak light detection [1,2,3]. Compared with traditional photomultiplier tube, APD is preferred to be used in harsh environments due to its superiority in terms of low power consumption, long lifetime, and insensitivity to magnetic field [4,5]. Based on the different material band gap, the Si- and InP-based APDs as commercial devices have been widely used in detection of visible and near-infrared lights, respectively [6,7]. For example, Si-based APDs are frequently used in space lidars [8,9] for laser ranging and are used as scintillation light detector for particle detection [10,11]. The InP-based APDs operation within near-infrared wavelength range has become a preferred choice for deep space optical communications [12,13]. In the above space and nuclear missions, APDs are exposed to irradiation environments and are expected to have strong radiation tolerances. 

The APD works depend on the carrier impact ionization under large electric field [14], where the high requirement on semiconductor quality means it may be sensitive to radiation damage. Especially for neutron irradiation, the introduced point defects or even disordered regions will lead to defect-related energy levels in the band gap [15,16]. The formed deep level defects serving as the carrier generation or recombination centers will have further influence on photodetector carrier collection and dark current [17,18]. Moreover, the APD operation in Geiger mode should not only consider carrier collection, but also impact ionization process [19], so the irradiation damage would be more complex than conventional photodetector. M. Musacci et al. reported that Si-based APDs exhibited a large increase of dark count rates but without substantial change in breakdown voltage after exposure to neutron fluences of 1 × 10^10^ cm^−2^ and 1 × 10^11^ cm^−2^ [20]. Richard D. H. et al. found that InP-based APDs demonstrated extreme sensitivity to displacement damage since they become unusable after being irradiated by a relatively small proton fluence of 7.5 × 10^9^ cm^−2^ [21]. Becker H. N. et al. pointed out that the structural complexity of InP-based APDs makes it is difficult to predict their radiation response based on analysis of the InGaAs absorption region alone, and carrier removal should be taken into account at very high fluence [22]. Recently, our group reported the effect of fast neutron irradiation on Si- and GaN-based APDs, whose gain and dark currents were analyzed in detail for the devices before reach the breakdown voltage (V_BD_) [23]. The results show that GaN-based APD has stronger radiation resistance than Si-based counterparts since their dark current and gain remain almost the same after being irradiated by a high neutron fluence of 1 × 10^14^ cm^−2^. The current research status indicates that little work has focused on the breakdown characteristic of APDs under different neutron fluences. Meanwhile, the Si- and InP-based APDs as the most used weak light detectors still lack a comparison in terms of radiation resistance.

In this paper, the neutron irradiation effects on avalanche breakdown characteristic of Si- and InP-based APDs are investigated. Both the two types of APDs show the increased dark current and the decreased gain after irradiation. However, there appears some obvious differences in the change of breakdown voltage and the degree to which the dark current is increased between the irradiated Si- and InP-based APDs. Combined with the APD working principle and the mechanism of neutron irradiation on semiconductors, we point out that whether the multiplication layer is doped will have a significant effect on APD radiation resistance. The results enrich the understanding of the effect of neutron irradiation on Si- and InP-based APDs.

## 2. Experiment

**Figure 1:**
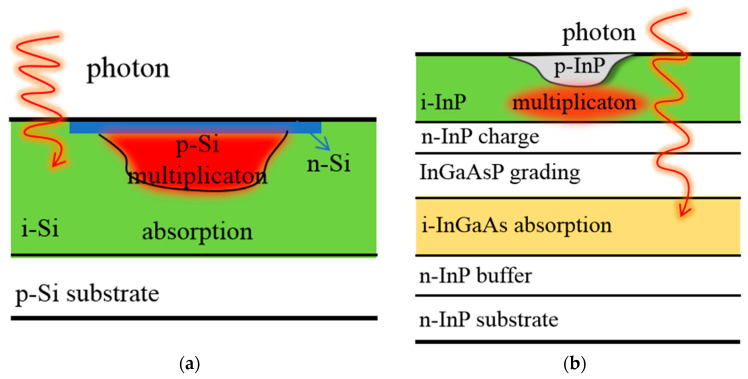
Schematic diagrams of a (**a**) Si-based APD and an (**b**) InP-based APD.

The Si- and InP-based APDs for neutron irradiation were obtained from commercial devices, with the structure schematically illustrated in Figure 1. The Si- and InP-based APDs are of planar geometry and are based on the structure of separate absorption and multiplication (SAM) regions. In the SAM structure, it is beneficial to optimize the electric field distribution in APDs to avoid premature breakdown, in which only one type of carrier (electrons or holes) triggers an avalanche. From the bottom to the top, the Si-based APDs include p-Si substrate, i-Si absorption layer, p-Si multiplication layer, and n-Si top contact layer. The multiplication layer and top contact layer are doped by boron ion and phosphorus ion implantation, respectively. For InP-based APDs, we commonly use the lattice-matched In_0.53_Ga_0.47_As as absorption layer and InP as multiplication layer since tunneling in In_0.53_Ga_0.47_As material easily occurs under high electric field. In addition, a suitable quaternary grading layer of InGaAsP and a thin layer of n-InP are sandwiched between the absorption layer and multiplication layer to smooth the large valence band discontinuity and adjust the electric field distribution, respectively [24]. 

The irradiation experiment was carried out at the Chinese Fast Burst Reactor-II (CFBR-II) of the Institute of Nuclear Physics and Chemistry, China Academy of Engineering Physics, with neutron energy controlled to ~1.2 MeV. The APDs under test were fixed to the inner side of some Al shells with different radius. As the radius increases, irradiation fluence will decrease. The Si- and InP-based APDs were divided into three groups to be irradiated by the neutron fluences of 1.0 × 10^12^ cm^−2^, 1.0 × 10^13^ cm^−2^, and 1.0 × 10^14^ cm^−2^, respectively. The three devices chosen from each type of APD have almost identical performance. All the irradiated devices were left for two weeks to ensure that radioactivity reduced to safety levels. The reverse current-voltage (I-V) characteristic was measured by a Keithley 2636B semiconductor analyzer. For the characterization of light response, incident monochromatic light was achieved by the combination of a monochromator and a Xe lamp or a bromine-tungsten lamp to cover the visible and near-infrared wavelength range.

## 3. Results and Discussions

**Figure 2:**
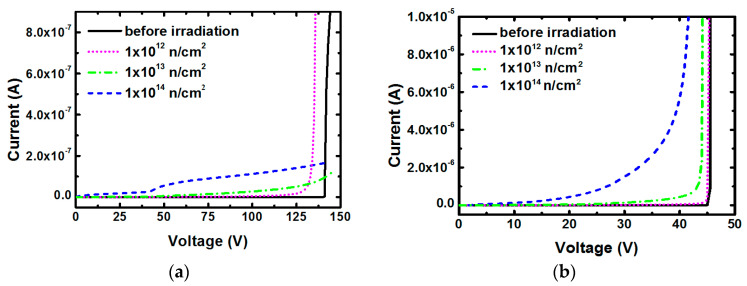
Reverse dark I-V characteristic of the (**a**) Si-based APDs and (**b**) InP-based APDs before and after neutron irradiation.

The dark current and responsivity are the two important characteristics of photodetectors that should be addressed since they have a direct effect on detection sensitivity. Dark current can be measured directly in the dark environment, while responsivity is determined by the photo-generate current and the incident light power. For Geiger-mode APDs, the photo-generate current is further influenced by multiplication gain and the applied voltage. In actuality, the APD responsivity (R) and gain (g) have a linear relationship as $R=\frac{\lambda q}{hc}\eta g$, where λ is the signal wavelength, c is the vacuum speed of light, q is the elementary charge, h is Planck’s constant, and η is the absorption efficiency [25]. Therefore, we will put the emphasis on irradiation effects of APDs dark current, multiplication gain and breakdown voltage. Figure 2 shows the dark I-V characteristic of (a) Si-based APDs and (b) InP-based APDs before and after irradiation. Before irradiation, both the two types of APDs exhibit low dark current in the reverse operating mode until the onset of the breakdown voltage. Sharp increase of dark currents is further observed as the bias voltages reach to above breakdown point, indicating that the tested devices have pretty good avalanche properties. Those two types of APDs have different breakdown voltages at ~141 V and ~45 V, respectively, for Si- and InP-based APDs. The electric field in multiplication layer directly determines the APD breakdown voltage, which is generally influenced by the breakdown characteristic of material itself, the thickness and doping concentration of each functional layers. In our opinion, the main reason for higher breakdown voltage of Si-based APD is probably due to its thicker multiplication layer with several micrometers [26], while the multiplication layer of InP-based APD is usually designed to be only a few hundred nanometers to avoid causing tunneling effect in InGaAs absorption layer under large breakdown voltage [27]. After irradiation, the dark currents of each type of APD show different neutron responses. For Si-based APDs, soft-breakdown appears at the minimum neutron fluence of 1.0 × 10^12^ cm^−2^ as the dark currents gradually increase with the increase of reverse voltage and still keep a steep ascent when the applied voltage higher than ~133 V. In this state, the breakdown voltage is reduced by ~8 V compared to the unirradiated devices. Under the low reverse voltage range, the dark current increases with the increase of neutron fluence. When the neutron fluence increases to 1.0 × 10^13^ cm^−2^ and 1.0 × 10^14^ cm^−2^, a loss of avalanche properties is observed since the dark currents never show sharp increase. As InP-based APD irradiated by a neutron fluence of 1.0 × 10^12^ cm^−2^, the dark current still retains a very sharp behavior, almost the same as the unirradiated device, indicating that it has little effect on APDs breakdown mechanism. Soft-breakdown is observed for the InP-based APDs irradiated by higher fluences of 1.0 × 10^13^ cm^−2^ and 1.0 × 10^14^ cm^−2^. The APD exhibits a huge increased leakage current at reverse bias conditions greater than 20 V when irradiated to a fluence of 1.0 × 10^14^ cm^−2^. For the fluence of 1.0 × 10^13^ cm^−2^, a smaller soft breakdown voltage of ~43.5 V is observed in comparison with the unirradiated device.

**Figure 3:**
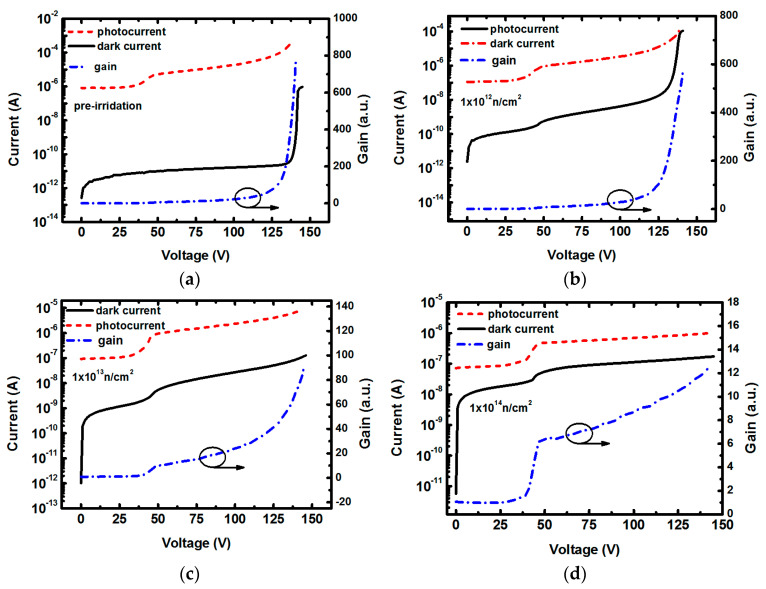
Reverse I-V characteristic of the Si-based APDs in dark and under illumination with incident light wavelength of 700 nm. The right-hand axis indicates the gain. (**a**) pre-irradiation; (**b**), (**c**), (**d**) under neutron fluences of 1.0 × 10^12^ cm^−2^, 1.0 × 10^13^ cm^−2^, 1.0 × 10^14^ cm^−2^, respectively.

The reverse I-V curves of Si-based APDs in dark and under illumination are shown in Figure 3, in which the ordinates use the logarithmic coordinate to clearly show the details of the curves. The wavelength of illumination light is filtered to 700 nm. Under illumination, the current of unirradiated Si-based APD arises about six orders of magnitude compared to that in dark condition, demonstrating obvious photo response. The Si-based APD irradiated under a neutron fluence of 1.0 × 10^12^ cm^−2^ still exhibits a pronounced photo response with current increase by about three orders of magnitude. Although light response can be observed for the APDs after higher irradiation fluences of 1.0 × 10^13^ cm^−2^ and 1.0 × 10^14^ cm^−2^, the increase of dark current greatly reduces the detection sensitivity. In Figure 3, we also show the bias-dependent gain characteristic in right-hand axis, in which the gain values are calculated from the equation $M_{ph}=\frac{I_{ML}-I_{MD}}{I_{L}-I_{D}}$ [28]. The $I_{ML}$ and $I_{MD}$ are the multiplied light and dark currents, while $I_{L}$ and $I_{D}$ are the un-multiplied light and dark currents, respectively. All the Si-based APDs photocurrent show a small step under the bias voltage of ~40 V, which we can identify the so-called “punch-through” voltage of SMA structure [23]. From that point, a further increase in bias voltage leads to an increase of the electric field primarily in the multiplication layer, and impact ionization happens only for the applied voltage higher than this punch-through point [29]. So, the un-multiplied light and dark currents are chosen from the average current value of the voltage range below 40 V. For the Si-based APD before irradiation, the gain curve exhibits a rapid increase to the maximum value, as high as ~800 when reaching the breakdown voltage, which is almost consistent with the variation trend of dark current. For the irradiated Si-APDs, the peak gain decreases with the increase of neutron fluence. The APD still has a peak gain excess ~500 and holds a relatively steep ascending feature after irradiated by the minimal fluence of 1.0 × 10^12^ cm^−2^. With the further increase of neutron fluence, the steep ascending feature around breakdown voltage cannot be observed and the highest gain values reduce to less than 100 and 15 under the irradiation fluences of 1.0 × 10^13^ cm^−2^ and 1.0 × 10^14^ cm^−2^, respectively. 

**Figure 4:**
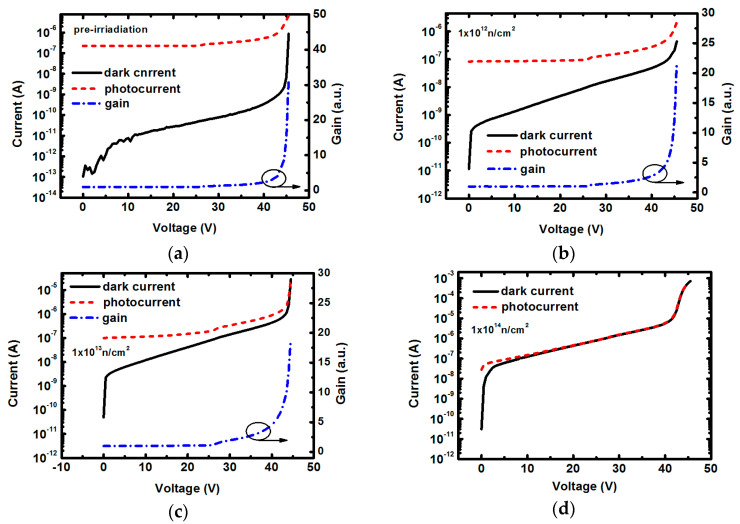
Reverse I-V characteristic of the InP-based APDs in dark and under illumination with incident light wavelength of 1550 nm. The right-hand axis indicates the gain. (**a**) pre-irradiation; (**b**), (**c**), (**d**) under neutron fluences of 1.0 × 10^12^ cm^−2^, 1.0 × 10^13^ cm^−2^, 1.0 × 10^14^ cm^−2^, respectively.

Similar to Si-based APDs, the reverse I-V curves of InP-based APDs in dark and under illumination are shown in Figure 4, in which the right-hand axis represents the gain value. Illumination light is controlled to the monochrome wavelength of ~1550 nm, located in the optical communication band. Under illumination, the unirradiated InP-based APD demonstrates strong photo response as the current increase by more than three orders of magnitude compared with the dark situation. Different from the dark current which increases obviously with the increase of neutron fluence, the photocurrent exhibits only a slight drop after neutron irradiation. After being irradiated by the highest neutron fluence of 1.0 × 10^14^ cm^−2^, the APD dark current and light current almost coincide with each other, indicating that the device have lost detection capability. A very small step of photocurrent is observed around punch-through voltage of ~25 V, below which can be considered as un-multiplied light and dark currents. Based on this, we calculate the APD bias-dependent gain characteristics. Before neutron irradiation, the APD gain shows a very rapid increase with peak value excesses ~30 when the applied voltage beyond breakdown point. Compared to the several hundreds of gain of Si-based APDs, InP-based APDs hold relatively low gain due to the different device material and structure with smaller operation voltage. For the InP-based APDs irradiated by neutron fluences of 1.0 × 10^12^ cm^−2^ and 1.0 × 10^13^ cm^−2^, the gain values still show a fast increase above breakdown voltage, but the steepness weakens with the increase of fluence. The highest gain reduces to less than ~20 under the neutron fluence of 1.0 × 10^13^ cm^−2^. We do not show the gain curve at higher fluence of 1.0 × 10^14^ cm^−2^ as the APD photo response becomes too low to be observable.

The above results indicate that neutron irradiation will lead to the increase of dark current and the decrease of gain for both the Si- and InP-based APDs, but there still exist some obvious differences. The Si-based APDs showed soft breakdown with a breakdown voltage reduced by ~8 V after irradiated by neutron fluence of 1.0 × 10^12^ cm^−2^. Meanwhile, the light response and gain can still be observed even under the highest irradiation fluence of 1.0 × 10^14^ cm^−2^. While for the InP-based APDs, very similar avalanche characteristic with the unirradiated devices was obtained under the neutron fluence of 1.0 × 10^12^ cm^−2^, and only a higher fluence of 1.0 × 10^13^ cm^−2^ caused soft breakdown and a small change in breakdown voltage. However, the light response can hardly be observed as the neutron fluence is further increased to 1.0 × 10^14^ cm^−2^. The observed results of the two types of APDs will be compared and analyzed in combination with the APD working principle and the semiconductor irradiation mechanism. In general, neutron irradiation on semiconductor materials mainly produces displacement damage, which removes atoms from the lattice position and results in vacancy or interstitial. Those neutron induced imperfect structures reflect the energy band to form energy levels in material band gap, acting as carrier capture centers or as generation centers according to the irradiation fluence and doping amount. Carrier capture mainly happens in doped materials and becomes more obvious with the increase of irradiation fluence. The neutron induced carrier removing will probably change the internal electric field distribution of APD. Introducing carrier generation centers will produce new carriers and thus cause additional dark current noise for APD. The amount of neutron induced dark current is mainly related to the material band gap and the irradiation fluence. In our experiment, the dark current of Si- and InP-based APDs increases almost linearly with the increase of irradiation fluence when the applied voltage lower than the breakdown point, confirming that the change of device performance is indeed caused by displacement damage. As the light absorption is a band-to-band process which is almost unchanged by irradiation to this level, the photocurrents of two types of APDs change little with the change of neutron fluence. 

**Figure 5:**
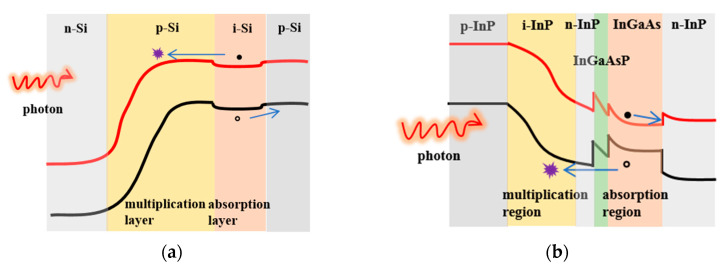
The schematic band diagrams of a (**a**) Si-based APD and an (**b**) InP-based APD, with the transportation process of photo-generated carriers is also shown.

Figure 5 shows the band structures of Si- and InP-based APDs, in which the carrier transportation process is also presented. For Si-based APDs, the incident photons enter into the device and reach the intrinsic absorption layer to generate the non-equilibrium electrons and holes. The photo-generate holes move towards the p-Si substrate to form a hole photocurrent, while the photo-generate electrons move to the opposite direction to trigger impact ionization in the multiplication layer and form the multiplied photocurrent. The InP-based APD has a more complex structure than that of Si-based APD, in which the lattice-matched In_0.53_Ga_0.47_As and InP are used as the absorption and multiplication layers, respectively. Moreover, there is also a charge layer and a grading layer sandwiched between absorption and multiplication layers to adjust the electric field distribution and smooth the band discontinuity, respectively. Absorbed photons create electron-hole pairs in the In_0.53_Ga_0.47_As material, and then the photo-generated carriers are swept via drift toward opposite directions. The photo-generated electrons move towards the n-InP substrate to form the electron photocurrent, while the photo-generate holes create additional electron-hole pairs in the multiplication layer to start the chain reaction of avalanche multiplication. 

InP-based APDs irradiated by the neutron fluence higher than 1.0×10^13^ cm^−2^ lead to a significantly increased reverse current prior to device breakdown, suggesting that the neutron induced carrier generation centers in the In_0.53_Ga_0.47_As absorption material play an important role in influencing dark current [22]. Considering that the increased dark current from the absorption layer will also enter into the multiplication layer to be amplified and irradiation has a small effect on photocurrent, the InP-based APD demonstrates almost unobservable light response under the highest fluence of 1.0 × 10^14^ cm^−2^. Another possible reason for unobservable light response is that InP-based APDs use holes to transport from absorption layer into multiplication layer to trigger impact ionization, whose transportation efficiency will be smaller than that of Si-based APDs with impact ionization triggered by electrons. This reason can also explain why avalanche gain still would be observed in Si-based APDs even after highest irradiation fluence of 1.0 × 10^14^ cm^−2^. For Si-based APDs after irradiation, the increase of dark current can be mainly attributed to the introduction of carrier generation centers in the depletion region. Theoretically, higher neutron fluence will introduce more generation centers in the depletion region, as a result, larger dark current will be observed as well. However, the Si-based APDs do not show a rapid increase of dark current around breakdown voltage after being irradiated by higher fluences of 1.0 × 10^13^ cm^−2^ and 1.0 × 10^14^ cm^−2^. The loss of the avalanche characteristic makes the current lower than the corresponding values before irradiation. In addition to introducing the generation center, another significant irradiation effect is the change in the effective doping concentration by carrier removal, especially for doped materials. We infer that the probable reason for this is that the Si-based APDs use doped material as multiplication layer. Hence, the neutron induced carrier capture effect becomes non-ignorable. Carrier capture could remove the intrinsic and photo-generated carriers, leading to the change of the electric field distribution away from the optimal state. As a result, the Si-based APDs are no longer able to launch impact ionization with high efficiency after being irradiated under the high fluences of 1.0 × 10^13^ cm^−2^ and 1.0 × 10^14^ cm^−2^. After neutron irradiation, the soft breakdown voltage of Si-based APDs shows a more significant change than InP-based APDs and the Si-based APDs exhibit soft breakdown earlier than InP-based APDs. The increase of neutron fluence can be also entirely ascribed to the change of electric field distribution. Based on the above analysis, it could be concluded that whether the multiplication layer is doped will have a great influence on the APD neutron irradiation hardness. 

## 4. Conclusions

In conclusion, the effects of fast neutron irradiation on the avalanche breakdown characteristic of Si- and InP-based APDs have been investigated. With the increase of neutron fluence, both the two types of APDs show the increased dark current and the decreased peak gain. The increase of dark current can be attributed to the introduction of generation centers in the Si depletion region and In_0.53_Ga_0.47_As absorption layer for Si- and InP-based APDs, respectively. The Si-based APD exhibit soft breakdown at the minimum fluence of 1.0 × 10^12^ cm^−2^, and the avalanche breakdown characteristic disappears after being irradiated by higher neutron fluences. For InP-based APDs irradiated by a fluence of 1.0×10^12^ cm^−2^, the reverse breakdown characteristic still retains a sharp activation very similar to the unirradiated devices. The occurrence of soft breakdown is initiated with the higher fluence of 1.0 × 10^13^ cm^−2^. Neutron irradiation seems to have a greater effect on Si-based APDs since the occurrence of soft breakdown is initiated from a lower fluence and the breakdown voltage shows a larger change than InP-based APDs. The Si-based APD using p-doped Si as the multiplication layer is considered the main reason that the neutron induced recombination defects play an important role in influencing the APD electric field distribution. Our findings will be helpful to design the APD with strong radiation resistance.

## Data Availability

The data presented in this study are available in article here.
